# Effect of low-intensity long-duration ultrasound on the symptomatic relief of knee osteoarthritis: a randomized, placebo-controlled double-blind study

**DOI:** 10.1186/s13018-018-0965-0

**Published:** 2018-10-16

**Authors:** David O Draper, Dominic Klyve, Ralph Ortiz, Thomas M Best

**Affiliations:** 1 Department of Exercise Sciences, Brigham Young University, 106 SFH, Provo, UT USA; 2 Department of Mathematics, Central Washington University, Ellensburg, USA; 3 UHealth Sports Performance and Wellness Institute, University of Miami, Florida, USA; 4 Medical Pain Consultants, Dryden, Dryden USA

**Keywords:** Osteoarthritis, Pain, Low-intensity ultrasound, Knee, Long duration, Musculoskeletal, Sustained acoustic medicine

## Abstract

**Background:**

Wearable long-duration low-intensity ultrasound is an emerging non-invasive and non-narcotic therapy for the daily treatment of musculoskeletal pain. The aim of this randomized, double-blind, placebo-controlled study was to examine whether long-duration low-intensity ultrasound was effective in treating pain and improving function in patients with knee osteoarthritis.

**Methods:**

Ninety patients with moderate to severe knee pain and radiographically confirmed knee osteoarthritis (Kellgren-Lawrence grade I/II) were randomized for treatment with active (*n* = 55) or placebo (*n* = 35) devices applied daily to the treated knee. Investigators and subjects were blinded to treatment groups. Ultrasound (3 MHz, 0.132 W/cm^2^, 1.3 W) was applied with a wearable device for 4 h daily for 6 weeks, delivering 18,720 J per treatment. The primary outcome was change in pain intensity (numeric rating scale) assessed prior to intervention (baseline) and after 6 weeks. Secondary outcomes of functional change were measured at baseline and after 6 weeks using the Western Ontario McMaster Osteoarthritis Questionnaire (*n* = 84), along with range of motion (flexion, extension) and isometric muscle strength (flexion, extension and rotation) tests on the injured knee in a small pilot subset (*n* = 17).

**Results:**

The study had a 93% retention rate, and there were no significant differences between the groups regarding demographic variables or baseline outcome measures. Patients treated with active therapy observed a significant mean NRS pain reduction over the 6-week study of 1.96 points for active (*p* < 0.0001), compared with a 0.85 points reduction for placebo (*p* = 0.13). The functional score was also significantly improved by 505 points for the active group over the 311-point improvement for placebo group compared to baseline (*p* = 0.02). In the pilot subset evaluated, rotational strength increased from baseline to 6 weeks (3.2 N, *p* = 0.03); however, no other measures were significant.

**Conclusions:**

Long-duration low-intensity ultrasound significantly reduced pain and improved joint function in patients with moderate to severe osteoarthritis knee pain. The clinical findings suggest that ultrasound may be used as a conservative non-pharmaceutical and non-invasive treatment option for patients with knee osteoarthritis. Additional research is warranted on non-weight bearing joints of the musculoskeletal system as well as extended treatment time frames and follow-up.

**Trial registration:**

NCT02083861, registered 11 March 2014, https://clinicaltrials.gov/ct2/show/results/NCT02083861

## Background

Osteoarthritis (OA) is a serious and debilitating health problem affecting more than 27 million Americans and is a common work-related injury simulated by repetitive stresses [1]. Arthritis impacts nearly one in three adults between the ages of 45 and 65 and half of all adults over 65, with consistent effect across multiple races and ethnicities [2]. The number of OA patients is projected to increase as the population skews older and obesity rates rise. The disease is characterized by degeneration of articular cartilage and joint inflammation together with chronic pain, stiffness, swelling, and limited mobility. Chronic pain from OA significantly affects patients’ quality of life, work productivity, and is associated with comorbidities such as depression, anxiety, and sleep disturbance [1]. The disease can develop from trauma, overuse, and genetic factors in any joint of the body, but it is most commonly found in the knee, hip, spine, shoulders, and hands. Osteoarthritis exacts an enormous financial toll on the healthcare system, as the second most expensive condition for US hospitals, with aggregate costs of $14.8 billion in 2011 alone [3].

The most frequent treatment for OA is prescription painkillers and anti-inflammatory medications [4]. Serious health risks are associated with these medications, including addiction and increased risk for gastrointestinal, renal, and cardiovascular problems. Topical non-steroidal anti-inflammatory drugs (NSAIDs) have partially addressed this issue, but many people with OA do not get sufficient pain relief with NSAIDs alone [5, 6]. Intra-articular hyaluronic-acid injections provide an alternative but are not a consistent approach to pain relief and can be painful to administer to patients [7]. Although non-pharmaceutical therapies such as physical therapy, exercise, massage, therapeutic ultrasound, water aerobics, and others exist to treat the symptoms of OA, many patients lack the ability or resources to access these therapies leading to worse knee OA pain and progressive disability [8].

Ultrasound treatment has been utilized in the medical setting with minimal safety risks for the symptomatic management of OA pain for many years; however, the clinical efficacy of office-based ultrasound remains controversial. In 2010, a Cochrane review determined that ultrasound may provide clinical value for knee OA patients by reducing pain and improving function and quality of life and that larger-scale clinical trials were justified [9]. More recently, new clinical trials on OA and rheumatoid arthritis, along with multiple meta-analyses of the literature, have found statistical support indicating that consistent ultrasound treatment of OA symptoms is more effective than placebo controls [10]. Specifically, for knee OA pain, ten randomized controlled trials (645 patients) treated with ultrasound showed a positive effect over placebo on knee pain and a reduction in the Western Ontario and McMaster Universities Arthritis Index (WOMAC) score [11]. Current existing evidence suggests ultrasound administered daily is effective for OA symptomatic management [11]. Unfortunately, daily ultrasound treatment in the clinical setting is unrealistic for many patients and medical professionals.

The aim of this randomized double-blinded placebo-controlled study was to determine whether a wearable home-use long-duration daily low-intensity ultrasound therapy is an effective treatment option for patients with knee osteoarthritis symptoms*.* Our study utilized sustained acoustic medicine (SAM), a United States Food and Drug Administration approved (2013) prescription use wearable multi-hour ultrasound device to investigate the efficacy of long duration ultrasound in treating osteoarthritic pain and disability. Validated measures of OA pain, stiffness, function, and strength were used to determine effectiveness.

## Methods

This prospective randomized, double-blinded, placebo-controlled, study was conducted in the USA and registered with ClinicalTrials.gov identifier NCT02083861. Patients meeting inclusion criteria, and who successfully completed 2 weeks of baseline pain measures were randomized 3:2 for a 6-week treatment with active (*n* = 55) or placebo (*n* = 35) SAM device. Patients subsequently self-applied the respective treatment 4 h per day for 6 weeks to the lateral and medial arthritic knee. Measurements of pain were recorded in a daily patient diary, while functional measurements were completed during clinic visits. The study was approved by the institutional review board of Schulman Associates, and all patients provided informed consent to participate. The procedures followed were in accordance with the ethical standards of the responsible committee on human experimentation and with the Helsinki Declaration of 1975, as revised in 2000.

The study was conducted in the Central New York region of the USA between March 2014 and January 2015. Patient enrollment was accomplished through a referral from Cayuga Medical Center, the community hospital, to Medical Pain Consultants LLC, the affiliated ambulatory care practice in Dryden, NY. The practice served as the setting for enrollment, training on the use of the device, 2-week visits of the patients with research staff, and pre/post functional measurements. The patient’s home/work setting served as the setting at which the device was self-administered and where pain measurements were recorded.

Included patients were 35 to 80 years of age, reported moderate to severe knee OA pain negatively affecting their life, were radiographically-confirmed mild to moderate knee osteoarthritis (Kellgren-Lawrence (KL) grade I/II [12]) in one or both knees based on fixed-flexion x-ray radiological findings for osteophytes or joint space narrowing in any compartment in the previous 12 months, and reported average baseline pain score between 3 and 7 on the numeric rating scale (0–10 NRS) the week preceding enrollment. In cases of bilateral knee OA, the more painful knee was selected for treatment; if equal pain, a flip of a coin was used to select the knee for treatment.

Exclusion criteria included having presence of severe knee OA (KL grade III); having had a knee replacement, surgical intervention, or hyaluronic acid injection in the affected knee in the previous 6 months; being a non-ambulatory patient; being unable to self-apply the device to their knee; having current treatment with corticosteroids; or having had osteoarthritis develop secondary to a metabolic disorder.

### Baseline measurements and randomization

Pain scores were recorded three times each day in a paper diary during the 2-week baseline period (weeks 1–2). Upon completion of baseline data, patients were asked to meet with clinical site staff to review data entry into the diary. WOMAC scores were obtained at the end of the baseline period for all subjects continuing to the treatment protocol (*n* = 90). A small pilot cohort was assessed for clinic-administered range of motion and muscle strength at the end of the baseline period (*n* = 17). Then, patients were randomized using a computer-generated random number list into either the active group, in which they received an active wearable device, or the placebo group, in which they received a device that functioned and appeared like the active device but did not emit ultrasound energy (deactivated transducers provided by the manufacturer). Treatment arms were balanced by age, sex, and BMI. Treatment allocation was concealed from the clinic and research staff enrolling patients and performing data entry for analysis. Study patients, the investigator, research assistants, and study staff were blinded to the intervention.

### Intervention protocol

**Fig. 1:**
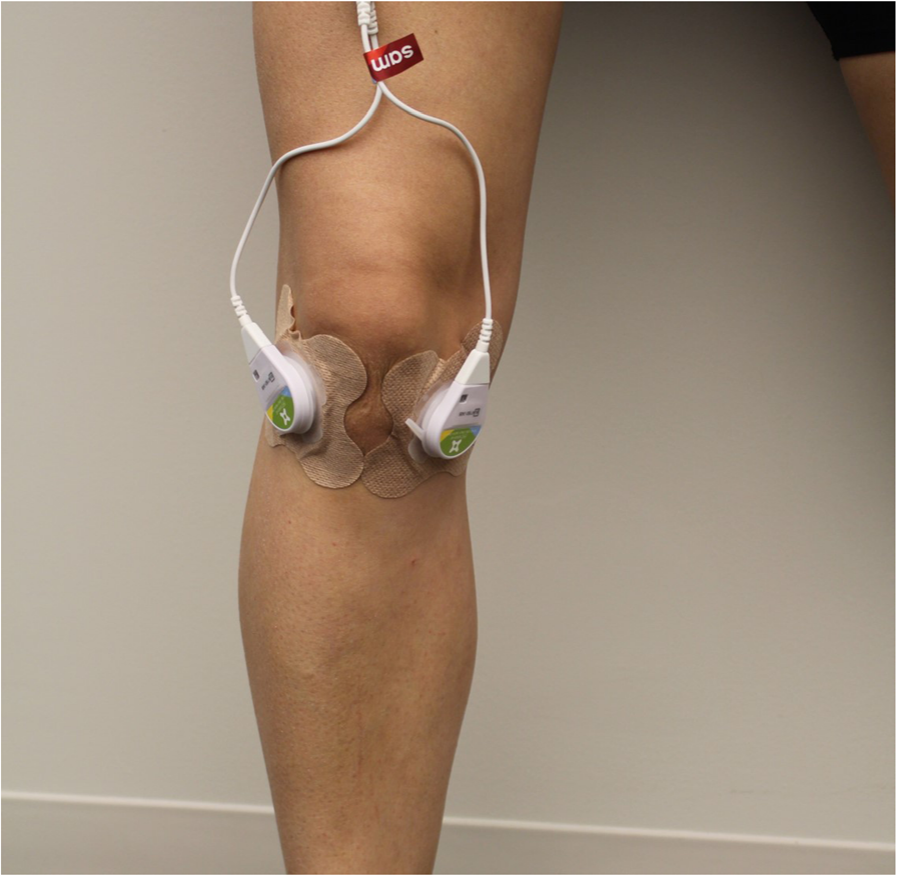
Wearable daily use ultrasound device. The wearable daily home-use long-duration low-intensity ultrasound device (SAM® Sport, ZetrOZ Systems LLC, Trumbull, CT) applied to the medial and lateral articulation points of the knee for the treatment of knee OA. The device is a prescription use only in the USA and has preconfigured ultrasound parameters of 3 MHz frequency and 1.3 W of energy for daily applied 4-h treatment

The long-duration low-intensity ultrasound treatment phase lasted 6 weeks (weeks 3–8). Patients were permitted to continue use of pain medications as long as those medications were maintained at a stable dose throughout the trial. Co-interventions were not assessed in this study. Ultrasound was self-administered in the home-setting with a wearable US Food and Drug Administration (FDA) approved (2013) class II prescription medical device SAM® Sport (ZetrOZ Systems, LLC, Trumbull, CT). The device was self-administered 4 h per day, 7 days per week for 6 weeks. The device operates at 3 MHz in continuous wave mode and delivers 1.3 W output power divided evenly across two transducers. The average ultrasonic intensity from each transducer is 132 mW/cm^2^ and the device delivers a total acoustic dose of 18,720 J of energy over the 4-h treatment period. The device is attached to the body with a disposable adhesive patch which comes pre-filled with ultrasonic coupling gel. At the clinic, patients were shown how to apply the transducers to the medial and lateral sides of the arthritic knee and set the medical devices treatment timer for 4 h of continuous ultrasound (Fig. 1). Patients were instructed to wear the device during normal daily activity and apply/remove the device when convenient with their daily schedule. Each patient randomized for treatment received one rechargeable device and three 40-packs of adhesive patches (120 individual units).

### Primary outcome

The primary outcome was weekly change in pain intensity relative to baseline through 6 weeks of therapy, as measured on the numeric rating scale (NRS) (0 = no pain, 10 = extreme pain). The NRS pain scale has been validated for consistency and reliability to assess pain associated with a variety of conditions, including OA [13]. Patients recorded their pain 4 h after applying the device during daily treatment.

### Secondary outcomes

Secondary outcome measures assessed were change in WOMAC score for pain stiffness and function, range of motion, and muscle strength assessed at baseline and the end of the study. Range of motion and strength measurements were acquired by trained clinic staff on a small pilot cohort (*n* = 17) of sequentially enrolled patients using computerized dual inclinometry range of motion and muscle tester equipment (JTECH Medical, Midvale, UT). To assess range of motion, one inclinometer was positioned on the quadricep muscle at mid femur and the other at mid-tibia. Patients were asked to lay flat on their back and lift their leg and flex at the knee to measure flexion. Patients were then asked to sit at the edge of the table and extend their leg to measure extension. To assess muscle strength, a manual muscle tester was used. Patients were asked to sit at the edge of the table with their knee at approximately 90 degrees. The manual muscle tester was positioned and held at anterior mid tibia while patients were asked to extend their leg to measure extension strength. To measure flexion strength, the muscle tester was held at anterior mid-tibia and the patient was asked to flex at the knee. Each range of motion and muscle strength test was repeated three times and the average was recorded.

### Follow-up

Once enrolled in the study, patients completed in-office visits on week 1 (patient enrollment and informed consent), week 3 (treatment randomization), week 5 (patient follow-up), week 7 (patient follow-up), and week 8 (study completion). During follow-up visits, the research staff reviewed the patient’s daily pain diary, addressed any questions the patient had about using the device or being involved in the study, and monitored for any adverse events (i.e., a serious unanticipated injury or death) or reactions (e.g., skin sensitivity, redness or burn) from the device.

### Statistical analysis

Chi-squared proportional assessment was used to assess gender demographics between groups, and *t* tests were used to analyze other demographic and outcome data. Data analysis was conducted in the R software environment for statistical computing (The R Foundation for Statistical Computing, Vienna, Austria). Data are expressed as means ± SDs (standard deviations). Statistical significance was achieved with *p* values less than 0.05.

## Results

### Enrollment and demographics of patient population

**Fig. 2:**
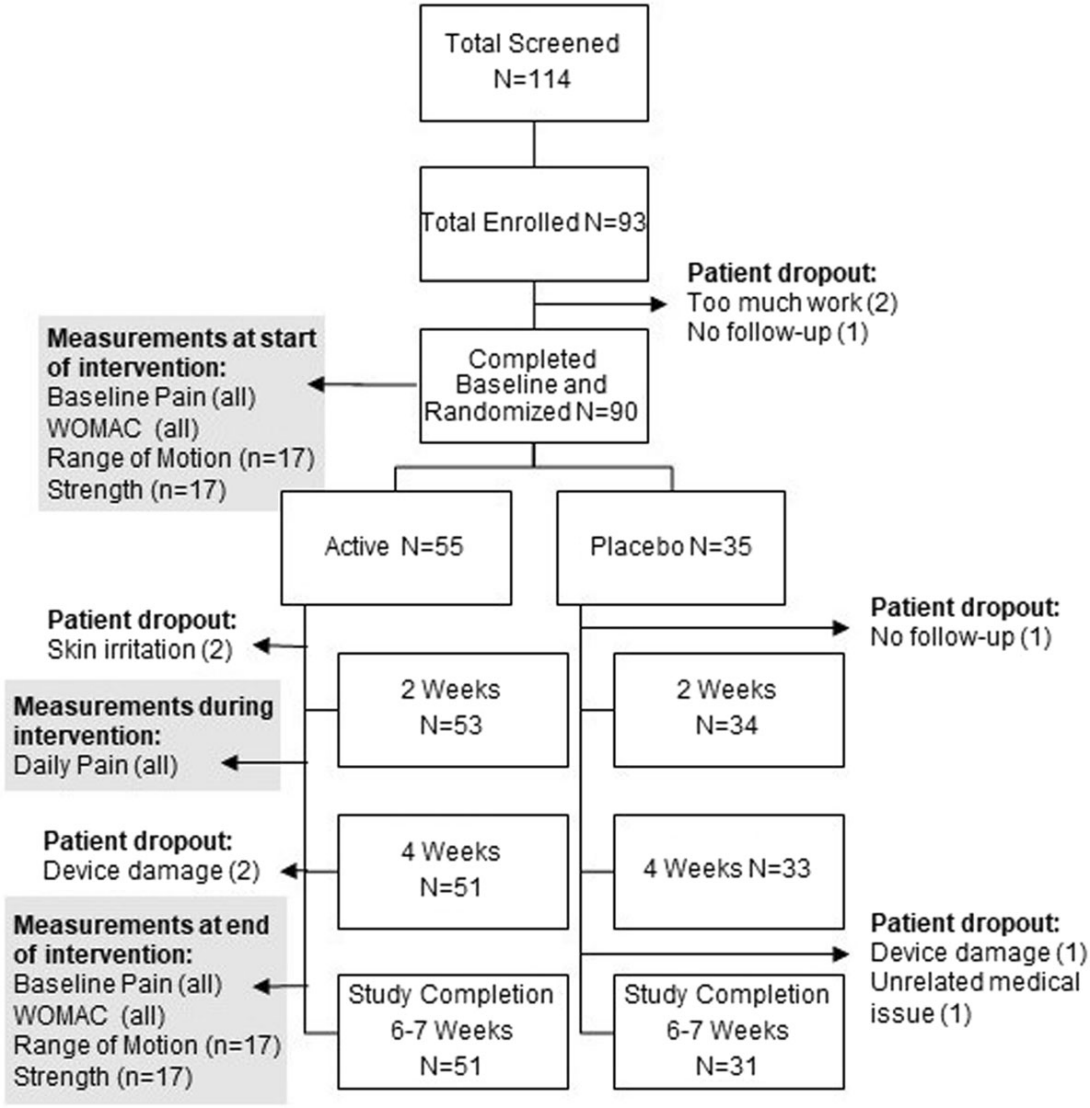
Study flow chart. Flow chart describing the progress of patients through the clinical trial on knee OA

A total of 114 patients were screened; 93 patients enrolled, and 90 patients were randomized into active or placebo groups (Fig. 2). Ten patients discontinued the study before it was completed: two indicated the study was too much work; two discontinued because of skin irritation (from the device); two were lost to follow-up; one discontinued for an unrelated medical issue; and three were lost from damaged medical equipment that was unable to be replaced. There were eight confirmed protocol violations, one in which the patient incorrectly applied the device on the unassigned knee that had a prior knee replacement, and seven patients who applied the device to both the assigned and unassigned knee. Of patients who completed the 2-week baseline assessment (*n* = 93), 90 were randomized into two ultrasound intervention groups (*n* = 55 active and *n* = 35 placebo). A total of *n* = 51 active and *n* = 33 placebo completed 6 weeks of therapy resulting in a 93% retention rate for the intervention.

The patient demographics for treatment intervention are shown in Table 1. After dropouts, treatment arms contained subjects with mean age 53.6 years active and 51.1 years placebo, gender 23/28 male/female active and 16/17 male/female placebo, and body mass index 34.9-BMI active and 34.5-BMI placebo. On average, patients had moderate pain at baseline 5.53-NRS active, 5.26-NRS placebo. No significant differences or trends were found between baseline pain and BMI for the groups (active versus placebo), or by gender. Approximately 88% (74 patients) of the study population was non-Hispanic Caucasian and 12% (10 patients) non-Hispanic African American. Enrolled patients were taking a median of four prescriptions during the course of the study. The most common pain medications were prescription NSAIDs and oxycodone, either in short-acting form (10 mg median dose) or sustained release (20 mg median dose). Cointervention results were not investigated in this study.

**Table 1:** Patient demographics of knee OA clinical trial

Patient demographic data			
Variable	Active ultrasound	Placebo ultrasound	*p*
*n*	51	31	
Sex (M/F)	23/28	16/17	0.115
Age, years	53.6 ± 8.9	51 ± 9.0	0.198
BMI	34.9 ± 8.85	34.5 ± 8.3	0.834

### Primary outcome measure of knee OA pain

There was a significant reduction of pain from baseline to the end of 6 weeks of therapy favoring activate low-intensity ultrasound treatment. Pain was reduced by 1.96 points for active, which was significant compared to 0.85-points reduction for placebo (1.11-point difference, *p* = 0.04) SAM device (Table 2). The active group changed significantly from baseline placebo group did not change significantly from baseline (*p* = 0.13).

**Table 2:** Pain on the NRS (0–10) scale reported daily in the patient diary after completion of 4-h ultrasound treatment. Mean ± SD

Primary outcome NRS data				
Week	Active	Placebo	Mean difference 95% CI	*p*
Baseline	5.53 (± 2.37)	5.26 (± 2.34)	0.27 (− 0.74 to 1.28)	0.59
2 weeks	3.61 (± 2.53)	4.48 (± 2.27)	− 0.87 (− 1.91 to 0.17)	0.10
4 weeks	3.29 (± 2.58)	4.26 (± 2.42)	− 0.97 (− 2.08 to 0.14)	0.08
6 weeks	3.57 (± 2.58)	4.41 (± 2.25)	− 0.84 (− 1.90 to 0.22)	0.11
NRS mean change from baseline 95% CI				
2 weeks	− 1.92 ± 2.39	− 0.78 ± 2.37	− 1.14 (− 2.18 to − 0.10)	0.03
	− 2.86 to − 0.98	− 1.89 to 0.32		
	*p* < 0.001	*p* = 0.16		
4 weeks	− 2.24 ± 2.47	− 1.00 ± 2.34	− 1.24 (− 2.31 to − 0.17)	0.02
	− 3.20 to 1.28	− 2.15 to 0.15		
	*p* < 0.001	*p* = 0.09		
6 weeks	− 1.96 ± 2.50	− 0.85 ± 2.41	− 1.11 (− 2.20 to − 0.02	0.04
	− 2.92 to 1.00	− 1.96 to 0.26		
	*p* < 0.001	*p* = 0.13		

### Secondary outcome measures of knee functional improvement

The WOMAC score measuring pain, stiffness, and function was significantly improved for active SAM by 505 points (*p* < 0.0001) and 311 points for placebo (*p* = 0.0002) after 6 weeks of intervention (Table 3). The improvement in the active group was significantly greater than the improvement of the placebo group (193-point difference, *p* = 0.02)**.** For WOMAC pain, the change of 107 points in the active group was significantly greater than that in the placebo group of 60.8 points (*p* = 0.02). For WOMAC stiffness, the change in the active group of 45 points was again significantly greater than that in the placebo group of 17 points (*p* = 0.002). For WOMAC function, the change in the active group of 352 points was significantly greater than the 220 points of the placebo group (*p* = 0.03).

**Table 3:** Secondary outcome measures, Western Ontario McMaster Osteoarthritis Questionnaire (WOMAC), and range of motion (ROM) and muscle strength measurements

Secondary outcome measurements							
Variable	Baseline			Endpoint		Endpoint group difference	
	Active	Placebo	*p*	Active	Placebo	Mean 95% CI	*p*
WOMAC Assessment							
*n*	55	35		51	31		
WOMAC pain	292 ± 89.1	276.5 ± 77.9	0.40	185 ± 103.2	215.7 ± 81.5	− 30.4	0.150
						− 72.4 to 11.6	
WOMAC pain mean change from baseline 95%CI				− 107.3 ± 97.5	− 60.8 ± 80.95	− 46.5 − 85.6 to − 7.4	*0.020*
				− 147.6 to − 66.8	− 100.3 to − 21.2		
				*p < 0.0001*	*p = 0.003*		
WOMAC stiffness	125 ± 35.8	123.5 ± 42.7	0.85	80.2 ± 41.1	106.4 ± 31.1	− 26.2 − 43.1 to 9.3	*0.003*
WOMAC stiffness mean change from baseline 95%CI				− 45 ± 39.0	− 17.1 ± 38.5	− 27.9 − 45.1 to − 10.7	*0.002*
				− 61.1 to − 28.9	− 36.0 to 1.9		
				*p < 0.0001*	*p = 0.080*		
WOMAC function	975 ± 272.2	974 ± 218.3	0.99	622.9 ± 336.7	754.8 ± 241.4	− 131.9 − 263.2 to 0.7	*0.049*
WOMAC function mean change from baseline 95%CI				− 352.3 ± 309.6	− 220.1 ± 233.6	− 132.2 − 250.5 to − 13.9	*0.029*
				− 480.7 to − 224	− 334.3 to − 105.9		
				*p < 0.0001*	*p = 0.0002*		
WOMAC total	1393 ± 377	1375 ± 299.4	0.81	888.4 ± 471	1063.7 ± 351.7	− 175.3 − 362.1 to 11.5	*0.066*
WOMAC total mean change from baseline 95%CI				− 504.6 ± 431.5	− 311.2 ± 331.33	−193.4 − 359.6 to − 27.2	*0.023*
				− 683.4 to − 325.7	− 473.4 to − 148.9		
				*p < 0.0001*	*p = 0.0002*		
ROM and strength assessments							
*n*	9	8		9	8		
ROM flexion (°)	41.5 ± 35.0	36.8 ± 28.5	0.76	35.1 ± 27.9	34.6 ± 25.1	0.570 − 27.1 to 28.2	0.97
ROM flexion mean change from baseline 95% CI				− 6.38 ± 33.6	− 2.22 ± 38.2	− 4.16 − 6.38 to − 2.22	0.79
				− 40.5 to 27.8	− 29.1 to 24.6		
				*p* = 0.69	*p* = 0.86		
ROM extension (°)	39.5 ± 35.9	37.1 ± 29.5	0.88	21.8 ± 28.0	31.6 ± 25.7	−9.81 −37.8 to 18.2	0.46
ROM extension mean change from baseline 95% CI				− 17.75 ± 34.1	− 5.56 ± 29.34	− 12.19 − 46.0 to 21.7	0.45
				− 52.5 to 17.0	− 33.2 to 22.1		
				*p* = 0.29	*p* = 0.68		
Strength flexion (*N*)	8.05 ± 3.64	10.4 ± 6.31	0.37	9.39 ± 2.92	11.4 ± 2.84	− 2.03 − 5.03 to 0.960	0.17
Strength flexion mean change from baseline 95% CI				1.34 ± 3.50	1.01 ± 5.19	0.33 − 5.26 to 5.92	0.90
				− 2.22 to 4.89	− 4.06 to 6.08		
				*p* = 0.43	*p* = 0.67		
Strength rotation (*N*)	6.06 ± 2.22	9.91 ± 5.52	0.086	9.28 ± 3.10	10.6 ± 3.09	− 1.31 − 4.52 to 1.90	0.40
Strength rotation mean change from baseline 95% CI				3.21 ± 2.86	0.68 ± 4.74	2.53 − 2.20 to 7.25	0.25
				0.294 to 6.06	− 3.89 to 5.25		
				*p = 0.03*	*p* = 0.75		
Strength extension (*N*)	7.79 ± 4.36	9.4 ± 5.29	0.51	9.70 ± 4.83	11.0 ± 2.81	− 1.30 − 5.59 to 2.99	0.50
Strength extension mean change from baseline 95% CI				1.91 ± 4.88	1.60 ± 4.49	0.31 − 4.78 to 5.40	0.90
				− 3.03 to 6.85	− 2.74 to 5.94		
				*p* = 0.42	*p* = 0.43		

*p* < 0.05 shown in italics

JTECH range of motion and strength measurements of the treated leg for active and placebo SAM are shown in Table 3. In total, five unique measurements where completed at baseline and after 6 weeks of intervention for *n* = 8 active and *n* = 9 placebo patients. These included flexion and extension along with muscle strength during neutral flexion, flexion-rotation, and extension. Independently, the range of motion and strength measurements were not significantly different, with the exception of muscle strength in flexion-rotation, with *p* values all above 0.1 for active versus placebo and comparing baseline to 6 weeks post-treatment. Strength in flexion-rotation increased 3.21 N in the active group after 6 weeks of treatment compared to baseline (*p* = 0.03).

## Discussion

The wearable SAM device can be successfully administered for use in the home setting under the monitoring of a healthcare professional familiar with low-intensity ultrasound devices. This non-pharmacological therapy shows high patient compliance and significantly improves symptomatic management of patients with KL grade I/II knee OA. In this study, 84 of 90 patients completed the SAM intervention phase (93% retention). The six patients who did not complete the study was a result of damage to the device (3), skin irritation (2), and no follow-up (1). Active SAM significantly reduced knee pain on average by 1.96 points after the 6-week intervention period (*p* < 0.001). Additionally, pain, stiffness, and function (WOMAC) improved significantly for patients with KL grade I/II knee OA treated with active SAM over the placebo group (*p* = 0.02, *p* = 0.002, *p* = 0.029 respectively).

These results are consistent with ultrasound literature for the symptomatic treatment of OA [14]. In a randomized, placebo-controlled study of daily ultrasound to treat hip osteoarthritis for 2 weeks, a significant 1.2-point difference on the visual analogy scale was found [15]. Another placebo-controlled study of ultrasound on knee osteoarthritis found similar results with a 2.8-point decrease in pain on the NRS after ten treatment sessions, and a significant 1.7-point difference in pain score between active and placebo ultrasound [16]. These and additional clinical findings are similar to the results of this study supporting ultrasound treatment for OA when applied five or more times per week [17].

In this study, patients with knee OA had an average BMI > 30 and were not asked to reduce or eliminate their pain medications. The use of moderate levels of prescription pain may have reduced the main effect of the home intervention. Additionally, the patient population was primarily Caucasian and the study was conducted in a rural environment.

Limited patients were enrolled in the range of motion and strength pilot studies. The active SAM group consistently had larger improvement of individual outcome measures; however, small group sizes potentially limited the power of the statistical analysis. Future studies conducted on range of motion and strength should include larger sample sizes.

The use of low-intensity ultrasound in the home setting appears to be an emerging treatment strategy for the non-invasive management of knee OA. Using a double-blind RCT design, the wearable SAM device was studied on a patient population which had clinically meaningful benefit from this intervention. Patients were able to use the device anytime during the day.

Low-intensity long-duration ultrasound treatment of arthritis pain is a novel, low-risk and non-invasive treatment option. The SAM device used in this study costs $4400 for 6 weeks of daily treatment, which is approximately half the cost of other low-intensity ultrasound devices for daily patient treatment. The SAM device used in this study permits the patient to set only the treatment time (1 to 4 h), reducing the need for intensive training of the patient. Furthermore, the wearable, patient-applied, multi-hour treatment does not interfere with the normal daily activity of the patient.

There are several similarities and differences worth noting between the wearable SAM device and other low-intensity ultrasound devices. The frequency and power of operation are within the same clinically utilized range of low-intensity ultrasound: between 1 to 3 MHz and 0.1 to 1.3 W. However, the total energy delivered by SAM was 18,720 J per 4-h treatment in this study, which is 133 times greater than most low-intensity therapy sessions which deliver 140 J per 20-min treatment. Monitoring of SAM device use and adherence to the study protocol was accomplished through in-office visits every 2 weeks. Wearable SAM ultrasound was well tolerated by the patients. In approximately 3528 unique treatment sessions with the device, two skin irritations were reported (less than 0.1%), and no significant safety risks or adverse events found.

The SAM device is not the only prescription (1 to 3 MHz) wearable low-intensity ultrasound therapy device available in the USA. Another device that provides 20 min of daily low-intensity ultrasound at 1.5 MHZ (Exogen®, Bioventus LLC, Durham, NC) was approved by the FDA in 1994. The use of wearable ultrasound may be considered as a non-pharmaceutical and non-invasive home-use treatment option for patients with moderate to severe osteoarthritis pain. The intervention could be particularly meaningful for arthritis patients who are not candidates or exploring non-invasive alternative options to prescription NSAIDs and narcotics, viscosupplements, or surgical procedures.

Future research on daily applied long-duration ultrasound shows promise to treat not only symptomology of osteoarthritis but perhaps the disease progression itself following joint injury or age-related degradation [18, 19]. The investigation into wearable low-intensity ultrasound treatment for various disease progression states and phenotypes of OA would yield insight into particular subsets of the OA population most likely to benefit from this treatment. Laboratory research with animal models of OA shows that ultrasound enhances expression of type II collagen, along with improved chondrocyte cell morphology and matrix integrity [20]. Whether or not these findings could be replicated in humans remains unknown. Ultrasound treatment in early-stage OA progression has significantly reduced the disease severity [21], and ultrasound treatment may also resolve arthritis-associated synovitis by mechanistically enhancing the phagocytosis of macrophages [22]. These encouraging data on the potential slowing of disease progression and the home-based ultrasound treatment strategy warrant further study. Here, the intervention period of 6 weeks was appropriate to capture meaningful changes in self-reported pain using the wearable low-intensity ultrasound device. Future research will investigate the long-term outcomes of this treatment option. Such research could provide significant healthcare benefit and perhaps change the way knee OA is managed early in the patient care continuum.

## Conclusions

Long-duration low-intensity ultrasound significantly reduced pain and improved joint function in patients with moderate to severe osteoarthritis knee pain. WOMAC scores were significantly reduced in the active ultrasound group including assessments for joint function. ROM and strength data collection limited the statistical power of findings here, and larger sample sizes may corroborate with WOMAC functional assessments. The clinical findings suggest that low-intensity ultrasound may be used as a conservative treatment option for patients with knee osteoarthritis. Additional osteoarthritis studies are necessary to determine the efficacy and compliance of long duration ultrasound use in other joints such as the spine.

## Abbreviations

BMI: Body mass index
FDA: Food and Drug Administration
KL: Kellgren and Lawrence
NRS: Numeric rating scale
NSAIDs: Non-steroidal anti-inflammatory drugs
OA: Osteoarthritis
ROM: Range of Motion
WOMAC: Western Ontario McMaster Osteoarthritis Questionnaire
